# PD-L1 expression associated with better response to EGFR tyrosine kinase inhibitors

**DOI:** 10.7497/j.issn.2095-3941.2015.0026

**Published:** 2015-06

**Authors:** Rafael Rosell, Ramón Palmero

**Affiliations:** ^1^Catalan Institute of Oncology, Hospital Germans Trias i Pujol, Badalona 08916, Spain; ^2^Germans Trias i Pujol Health Sciences Institute and Hospital, Campus Can Ruti, Badalona 08916, Spain; ^3^Instituto Oncológico Dr Rosell, Quiron Dexeus University Hospital, Barcelona 08028, Spain; ^4^Pangaea Biotech, Barcelona 08028, Spain; ^5^Molecular Oncology Research (MORe) Foundation, Barcelona 08028, Spain; ^6^Thoracic Oncology Unit, Department of Medical Oncology, Catalan Institute of Oncology, L’Hospitalet, Barcelona 08908, Spain

Cancer evades host immune surveillance by using immune checkpoints, which are inhibitory pathways crucial for maintaining self-tolerance^1^. Tumor cells express multiple inhibitory ligands, and tumor-infiltrating lymphocytes (TIL) express a variety of inhibitory receptors. Inhibitory receptors cytotoxic T-lymphocyte-associated protein 4 (CTLA-4) and programmed death-1 (PD-1)^2^ are the most studied immune checkpoint receptors. Activation of PD-1 and the programmed cell death ligand-1 (PD-L1) signal pathway result in the formation of an immunosuppressive tumor microenvironment, which causes tumor cells to escape organism immune surveillance. Tumor cells that escape immunosurveillance can become clinically detectable and can induce an immunosuppressive state through production of cytokines and growth factors, as well as by recruiting T-regulatory cells (T-regs) and myeloid-derived suppressor cells. The PD-1 receptor is a member of the immunoglobulin B7-CD28 family, is a negative regulator of T-lymphocyte activation, and can be expressed on TILs, similar to activated CD4^+^T, CD8^+^T, B, natural killer T, mononuclear cells, and dendritic cells. PD-L1 is expressed in many cancers, including non-small cell lung cancer (NSCLC). Immune cells play an important part in blocking the “cancer immunity cycle” by binding PD-1^1^. Inhibition of the CTLA-4 and PD-1 pathways has been shown to enhance intratumoral immune responses in numerous preclinical studies, and blockade of immune checkpoints has ushered in a new era in cancer treatment^1^.

Treatment strategies for NSCLC include chemotherapy regimens based on histology and targeted agents for patients who carry somatic activated oncogenes, such as epidermal growth factor receptor (*EGFR*) and translocated anaplastic lymphoma kinase (*ALK*). Majority of patients do not attain prolonged disease control, and 5-year survival rates remain low^3^. Increasing evidence has shown a relationship between PD-L1 expression and genetic alterations in oncogenes and tumor suppressor genes. For instance, deletion of *PTEN* leads to PD-L1 up-regulation in lung squamous cell carcinoma^4^. Moreover, patients with *EGFR*-mutant NSCLC may be susceptible to PD-1 blockade immunotherapy^5^. *EGFR*-mutant lung tumors inhibit antitumor immunity by activating the PD-1/PD-L1 pathway to suppress T-cell function and increase levels of proinflammatory cytokines; exposure to EGFR-TKIs leads to PD-L1 downregulation^5^. An anti-PD-1 antibody significantly reduces tumor growth and prolongs the survival of mice with EGFR-mutant adenocarcinoma^5^. Other oncogene-activated models may similarly drive immune escape.

A recent study in the *British Journal of Cancer* has reconfirmed that PD-L1 expression is correlated with *EGFR* mutations^6^. D’Incecco *et al*.^6^ examined PD-1 and PD-L1 expression via immunohistochemistry in a cohort of 125 NSCLC patients, 30 of whom where “triple negative” (wild-type *EGFR*, *ALK*, and *KRAS*) and the other 95 were *EGFR*-mutant, *KRAS*-mutant, or *ALK* translocated. All cases with moderate (+2) or strong staining (+3) in more than 5% of tumor cells^6^ were regarded as PD-1 or PD-L1 positive. The investigators identified different clinical and biological profiles of patients according to PD-1 and PD-L1 expression^6^. Patients with PD-1 positive tumors tended to be male and/or smokers with *KRAS*-mutant adenocarcinoma. By contrast, patients with PD-L1 positive tumors were more likely to be female and/or never or former smokers with *EGFR*-mutant or *ALK*-translocated adenocarcinoma histology^6^.

Until now, the results of the correlation between PD-1/PD-L1 expression and *EGFR* mutations remain controversial. Gettinger *et al*.^7^ reported that *EGFR* or *KRAS* mutations did not correlate with response rate to nivolumab for NSCLC patients. Some researchers found that activation of the EGFR pathway induced PD-L1 expression to help NSCLC tumors evade the antitumor immune response^5^^,^^8^. Mu *et al*.^9^ did not observe a significant correlation between PD-L1 expression and *EGFR*, *KRAS*, *BRAF*, or *ALK* status in stage I NSCLC patients. Similarly, Zhang *et al*.^10^ found no significant relationship between PD-L1 expression and mutational status of *EGFR* or *KRAS* in lung adenocarcinoma. Most recently, Ansen *et al*.^11^ reported that PD-L1 expression was associated with distinct genotypes of *EGFR*, *KRAS*, and *STK11* in the two most common histological NSCLC subtypes (i.e., adenocarcinoma and squamous cell carcinoma) in the 2014 ASCO Annual Meeting.

D’Incecco *et al*.^6^ observed that PD-1 positive status was significantly associated with the presence of *KRAS* mutations, whereas PD-L1 positive status was significantly associated with presence of *EGFR* mutations. Recent data presented by Rizvi *et al*.^12^ demonstrate that neoantigens created by nonsynonymous mutations may underlie the activity of PD-1 inhibition in NSCLC, that nonsynonymous mutation burden may be a predictive biomarker of response to anti-PD-1 therapy, and that immunotherapy may be beneficial for smoking-associated lung cancers. Pembrolizumab was more efficient in patients with a smoking-associated mutational signature (transversion-high tumors), which correlated with nonsynonymous mutation burden and a higher quantity of putative neoantigens^12^. Patients with a durable clinical response had a higher neoantigen burden than those without, suggesting that T-cell responses to neoantigens created by somatic mutations may underlie pembrolizumab activity in NSCLC^12^.

Besides smoking-related changes, the researchers were also able to identify other mutations present in lung cancer that may contribute to a high mutation burden and response to PD-1 inhibition. Specifically, they noted deleterious mutations in DNA repair and replication genes that have high mutation burden response and high response to pembrolizumab, such as *POLD1*, *POLE*, and *MSH2*. Some of these mutations occur in never-smokers with high mutational burden; this finding may explain why some never-smokers may also respond to therapy with PD-1 inhibitors^12^.

D’Incecco *et al*.^6^ found that patients with PD-L1 positive expression had higher sensitivity to EGFR-TKIs, longer time to progression (TTP), and better overall survival than PD-1 negative patients. Among 95 patients treated with gefitinib or erlotinib, sensitivity to TKIs was significantly correlated with PD-L1 expression, whereas tumor PD-1 expression did not seem significant in terms of response rate, TTP, and survival. Furthermore, among the 54 *EGFR*-mutant patients, TTP to EGFR TKI was significantly longer in PD-L1 positive than negative tumors^6^. Although PD-L1 is regarded as an immunosuppressive molecule, its expression is not necessarily synonymous with tumor immune evasion and may reflect an ongoing antitumor immune response that includes production of IFN-γ and other inflammatory factors. This finding is consistent with retrospective studies in NSCLC, where tumor PD-L1 expression has been shown to be a positive prognostic factor. Currently, the feasibility of PD-L1 expression level as a prognostic index has not been confirmed. Retrospective analysis has shown that overexpression of PD-L1 in NSCLC cells indicates high invasiveness and poor prognosis: Yang *et al*.^13^ reported that pulmonary adenocarcinoma patients with high expression of PD-L1 had longer recurrence-free survival than those with low expression of PD-L1. Velcheti *et al*.^14^ showed that patients with PD-L1 protein or mRNA overexpression had longer total survival not correlated with age, staging, or tissue type compared with patients with PD-L1 protein or mRNA under-expression.

D’Incecco *et al*.^6^ described PD-1 expression on tumor cells for the first time. Until now, the evidence points to PD-L1 being commonly up-regulated in NSCLC and PD-1 being expressed on the majority of TILs. This result explains the development of monoclonal antibodies against PD-L1 or PD-1. However, the authors did not examine PD-1 expression on CD8^+^ TILs or explore any correlation that may exist between PD-1-positive TILs and expression of PD-L1 on cancer cells.

A suitable test should be created to measure PD-L1 expression levels with established thresholds that can be used as a biomarker for anti-PD-1/PD-L1 therapies. A multitude of questions pertaining to companion predictive biomarkers to anti-PD-1/anti-PD-L1 therapies remain unanswered: Which PD-L1 antibody most accurately and reproducibly measures PD-L1 protein expression and predicts response to therapy? Which cutoff should be utilized to determine PD-L1 positivity/negativity? Should PD-L1 protein be measured in the tumor epithelium, stroma, or both? Should a different measure of PD-L1 expression, such as quantitative immunofluorescence or RNA, be used instead of standard immunohistochemistry protein methodology? Which additional components, such as TILs, PD-1, or PD-L2, play a role in predicting response? Currently, the various assays tend to be propriety to each of the groups developing the antibodies. Most assays examine PD-L1 staining on the tumor. Based on recent data from Herbst *et al*.^15^, some assays observe PD-L1 staining on immune infiltrate, including tumor and immune cells and the entire microenvironment. To date, we still do not know what antibodies will emerge or what the final cutoffs will be for a valid test measuring PD-L1 expression levels. Instead of using binary cutoffs to determine positivity/negativity, some researchers, including D’Incecco *et al*.^6^, have investigated quantitative measurements of PD-L1 expression. Quantitative measurement has proven difficult due to the apparent heterogeneity of PD-L1 expression, but whether a more quantifiable assay can better predict the response to anti-PD-1/anti-PD-L1 therapies remains unknown. Multiple companion predictive biomarkers that measure components in the PD-1/PD-L1 axis, TILs, and various stimulatory molecules will be required to predict response to immune therapies. Finally, the data from D’Incecco *et al*. suggest that *EGFR*-mutant NSCLC is highly eligible for PD-1/PD-L1 immunotherapy, and PD-L1 may represent a favorable biomarker candidate for response to EGFR-TKIs. If this finding is reconfirmed in prospective studies, then immune checkpoint blockade combination with EGFR TKIs could be a major step forward in improving outcomes of EGFR-mutant NSCLC patients.
